# RNA-Seq of Tumor-Educated Platelets Enables Blood-Based Pan-Cancer, Multiclass, and Molecular Pathway Cancer Diagnostics

**DOI:** 10.1016/j.ccell.2015.09.018

**Published:** 2015-11-09

**Authors:** Myron G. Best, Nik Sol, Irsan Kooi, Jihane Tannous, Bart A. Westerman, François Rustenburg, Pepijn Schellen, Heleen Verschueren, Edward Post, Jan Koster, Bauke Ylstra, Najim Ameziane, Josephine Dorsman, Egbert F. Smit, Henk M. Verheul, David P. Noske, Jaap C. Reijneveld, R. Jonas A. Nilsson, Bakhos A. Tannous, Pieter Wesseling, Thomas Wurdinger

**Affiliations:** 1 Department of Pathology, VU University Medical Center, Cancer Center Amsterdam, De Boelelaan 1117, 1081 HV Amsterdam, the Netherlands; 2 Department of Neurosurgery, VU University Medical Center, Cancer Center Amsterdam, De Boelelaan 1117, 1081 HV Amsterdam, the Netherlands; 3 Department of Neurology, VU University Medical Center, Cancer Center Amsterdam, De Boelelaan 1117, 1081 HV Amsterdam, the Netherlands; 4 Department of Clinical Genetics, VU University Medical Center, Cancer Center Amsterdam, De Boelelaan 1117, 1081 HV Amsterdam, the Netherlands; 5 Department of Neurology, Massachusetts General Hospital and Neuroscience Program, Harvard Medical School, 149 13th Street, Charlestown, MA 02129, USA; 6 thromboDx B.V., 1098 EA Amsterdam, the Netherlands; 7 Department of Oncogenomics, Academic Medical Center, Meibergdreef 9, 1105 AZ Amsterdam, the Netherlands; 8 Department of Pulmonary Diseases, VU University Medical Center, Cancer Center Amsterdam, De Boelelaan 1117, 1081 HV Amsterdam, the Netherlands; 9 Department of Medical Oncology, VU University Medical Center, Cancer Center Amsterdam, De Boelelaan 1117, 1081 HV Amsterdam, the Netherlands; 10 Department of Radiation Sciences, Oncology, Umeå University, 90185 Umeå, Sweden; 11 Department of Pathology, Radboud University Medical Center, 6500 HB Nijmegen, the Netherlands

## Abstract

Tumor-educated blood platelets (TEPs) are implicated as central players in the systemic and local responses to tumor growth, thereby altering their RNA profile. We determined the diagnostic potential of TEPs by mRNA sequencing of 283 platelet samples. We distinguished 228 patients with localized and metastasized tumors from 55 healthy individuals with 96% accuracy. Across six different tumor types, the location of the primary tumor was correctly identified with 71% accuracy. Also, MET or *HER2*-positive, and mutant *KRAS*, *EGFR*, or *PIK3CA* tumors were accurately distinguished using surrogate TEP mRNA profiles. Our results indicate that blood platelets provide a valuable platform for pan-cancer, multiclass cancer, and companion diagnostics, possibly enabling clinical advances in blood-based “liquid biopsies”.

## Highlights

• Tumors “educate” platelets (TEPs) by altering the platelet RNA profile
• TEPs provide a RNA biosource for pan-cancer, multiclass, and companion diagnostics
• TEP-based liquid biopsies may guide clinical diagnostics and therapy selection
• A total of 100–500 pg of total platelet RNA is sufficient for TEP-based diagnostics

## Graphical Abstract

**Figure:**
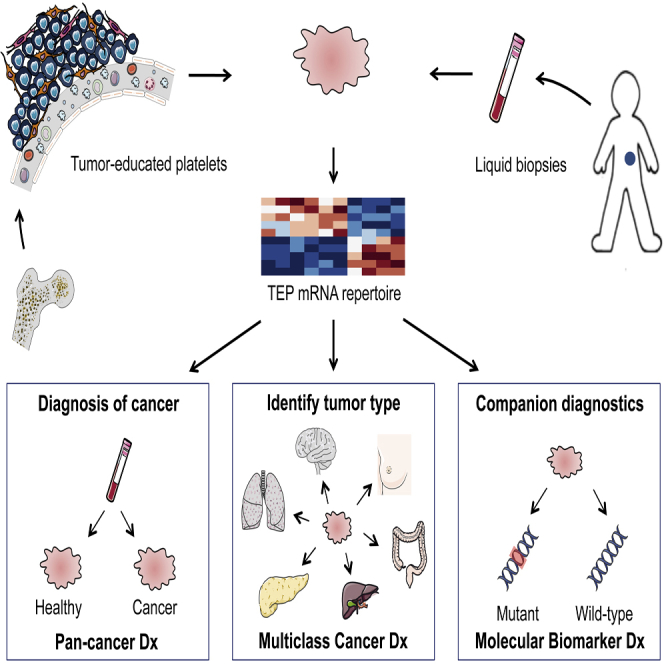

## Teaser

Best et al. show that mRNA sequencing of tumor-educated blood platelets distinguishes cancer patients from healthy individuals with 96% accuracy, differentiates between six primary tumor types of patients with 71% accuracy, and identifies several genetic alterations found in tumors.

## Significance

**Blood-based “liquid biopsies” provide a means for minimally invasive molecular diagnostics, overcoming limitations of tissue acquisition. Early detection of cancer, clinical cancer diagnostics, and companion diagnostics are regarded as important applications of liquid biopsies. Here, we report that mRNA profiles of tumor-educated blood platelets (TEPs) enable for pan-cancer, multiclass cancer, and companion diagnostics in both localized and metastasized cancer patients. The ability of TEPs to pinpoint the location of the primary tumor advances the use of liquid biopsies for cancer diagnostics. The results of this proof-of-principle study indicate that blood platelets are a potential all-in-one platform for blood-based cancer diagnostics, using the equivalent of one drop of blood.**

## Introduction

Cancer is primarily diagnosed by clinical presentation, radiology, biochemical tests, and pathological analysis of tumor tissue, increasingly supported by molecular diagnostic tests. Molecular profiling of tumor tissue samples has emerged as a potential cancer classifying method (Akbani et al., 2014, Golub et al., 1999, Han et al., 2014, Hoadley et al., 2014, Kandoth et al., 2013, Ramaswamy et al., 2001, Su et al., 2001). In order to overcome limitations of tissue acquisition, the use of blood-based liquid biopsies has been suggested (Alix-Panabières et al., 2012, Crowley et al., 2013, Haber and Velculescu, 2014). Several blood-based biosources are currently being evaluated as liquid biopsies, including plasma DNA (Bettegowda et al., 2014, Chan et al., 2013, Diehl et al., 2008, Murtaza et al., 2013, Newman et al., 2014, Thierry et al., 2014) and circulating tumor cells (Bidard et al., 2014, Dawson et al., 2013, Maheswaran et al., 2008, Rack et al., 2014). So far, implementation of liquid biopsies for early detection of cancer has been hampered by non-specificity of these biosources to pinpoint the nature of the primary tumor (Alix-Panabières and Pantel, 2014, Bettegowda et al., 2014).

**Figure 1:**
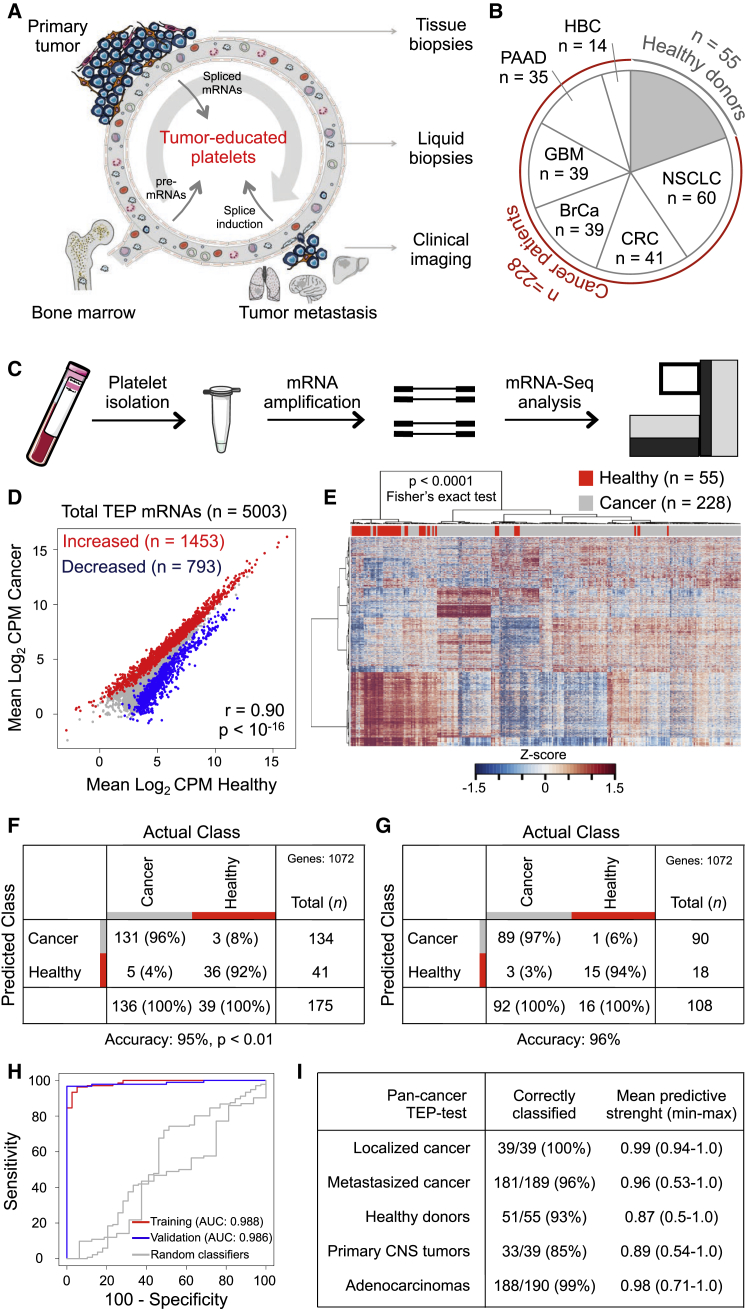
Tumor-Educated Platelet mRNA Profiling for Pan-Cancer Diagnostics (A) Schematic overview of tumor-educated platelets (TEPs) as biosource for liquid biopsies. (B) Number of platelet samples of healthy donors and patients with different types of cancer. (C) TEP mRNA sequencing (mRNA-seq) workflow, as starting from 6 ml EDTA-coated tubes, to platelet isolation, mRNA amplification, and sequencing. (D) Correlation plot of mRNAs detected in healthy donor (HD) platelets and cancer patients’ TEPs, including highlighted increased (red) and decreased (blue) TEP mRNAs. (E) Heatmap of unsupervised clustering of platelet mRNA profiles of healthy donors (red) and patients with cancer (gray). (F) Cross-table of pan-cancer SVM/LOOCV diagnostics of healthy donor subjects and patients with cancer in training cohort (n = 175). Indicated are sample numbers and detection rates in percentages. (G) Performance of pan-cancer SVM algorithm in validation cohort (n = 108). Indicated are sample numbers and detection rates in percentages. (H) ROC-curve of SVM diagnostics of training (red), validation (blue) cohort, and random classifiers, indicating the classification accuracies obtained by chance of the training and validation cohort (gray). (I) Total accuracy ratios of SVM classification in five subgroups, including corresponding predictive strengths. Genes, number of mRNAs included in training of the SVM algorithm. See also Figure S1 and Tables S1, S2, S3, and S4.

It has been reported that tumor-educated platelets (TEPs) may enable blood-based cancer diagnostics (Calverley et al., 2010, McAllister and Weinberg, 2014, Nilsson et al., 2011). Blood platelets—the second most-abundant cell type in peripheral blood—are circulating anucleated cell fragments that originate from megakaryocytes in bone marrow and are traditionally known for their role in hemostasis and initiation of wound healing (George, 2000, Leslie, 2010). More recently, platelets have emerged as central players in the systemic and local responses to tumor growth. Confrontation of platelets with tumor cells via transfer of tumor-associated biomolecules (“education”) is an emerging concept and results in the sequestration of such biomolecules (Klement et al., 2009, Kuznetsov et al., 2012, McAllister and Weinberg, 2014, Nilsson et al., 2011, Quail and Joyce, 2013). Moreover, external stimuli, such as activation of platelet surface receptors and lipopolysaccharide-mediated platelet activation (Denis et al., 2005, Rondina et al., 2011), induce specific splicing of pre-mRNAs in circulating platelets (Power et al., 2009, Rowley et al., 2011, Schubert et al., 2014). Platelets may also undergo queue-specific splice events in response to signals released by cancer cells and the tumor microenvironment—such as stromal and immune cells. The combination of specific splice events in response to external signals and the capacity of platelets to directly ingest (spliced) circulating mRNA can provide TEPs with a highly dynamic mRNA repertoire, with potential applicability to cancer diagnostics (Calverley et al., 2010, Nilsson et al., 2011) (Figure 1A). In this study, we characterize the platelet mRNA profiles of various cancer patients and healthy donors and investigate their potential for TEP-based pan-cancer, multiclass cancer, and companion diagnostics.

## Results

### mRNA Profiles of Tumor-Educated Platelets Are Distinct from Platelets of Healthy Individuals

We prospectively collected and isolated blood platelets from healthy donors (n = 55) and both treated and untreated patients with early, localized (n = 39) or advanced, metastatic cancer (n = 189) diagnosed by clinical presentation and pathological analysis of tumor tissue supported by molecular diagnostics tests. The patient cohort included six tumor types, i.e., non-small cell lung carcinoma (NSCLC, n = 60), colorectal cancer (CRC, n = 41), glioblastoma (GBM, n = 39), pancreatic cancer (PAAD, n = 35), hepatobiliary cancer (HBC, n = 14), and breast cancer (BrCa, n = 39) (Figure 1B; Table 1; Table S1). The cohort of healthy donors covered a wide range of ages (21–64 years old, Table 1).

Platelet purity was confirmed by morphological analysis of randomly selected and freshly isolated platelet samples (contamination is 1 to 5 nucleated cells per 10 million platelets, see Supplemental Experimental Procedures), and platelet RNA was isolated and evaluated for quality and quantity (Figure S1A). A total of 100–500 pg of platelet total RNA (the equivalent of purified platelets in less than one drop of blood) was used for SMARTer mRNA amplification and sequencing (Ramsköld et al., 2012) (Figures 1C and S1A). Platelet RNA sequencing yielded a mean read count of ∼22 million reads per sample. After selection of intron-spanning (spliced) RNA reads and exclusion of genes with low coverage (see Supplemental Experimental Procedures), we detected in platelets of healthy donors (n = 55) and localized and metastasized cancer patients (n = 228) 5,003 different protein coding and non-coding RNAs that were used for subsequent analyses. The obtained platelet RNA profiles correlated with previously reported mRNA profiles of platelets (Bray et al., 2013, Kissopoulou et al., 2013, Rowley et al., 2011, Simon et al., 2014) and megakaryocytes (Chen et al., 2014) and not with various non-related blood cell mRNA profiles (Hrdlickova et al., 2014) (Figure S1B). Furthermore, DAVID Gene Ontology (GO) analysis revealed that the detected RNAs are strongly enriched for transcripts associated with blood platelets (false discovery rate [FDR] < 10^−126^).

Among the 5,003 RNAs, we identified known platelet markers, such as B2M, PPBP, TMSB4X, PF4, and several long non-coding RNAs (e.g., MALAT1). A total of 1,453 out of 5,003 mRNAs were increased and 793 out of 5,003 mRNAs were decreased in TEPs as compared to platelet samples of healthy donors (FDR < 0.001), while presenting a strong correlation between these platelet mRNA profiles (r = 0.90, Pearson correlation) (Figure 1D). Unsupervised hierarchical clustering based on the differentially detected platelet mRNAs distinguished two sample groups with minor overlap (Figure 1E; Table S2). DAVID GO analysis revealed that the increased TEP mRNAs were enriched for biological processes such as vesicle-mediated transport and the cytoskeletal protein binding while decreased mRNAs were strongly involved in RNA processing and splicing (Table S3). A correlative analysis of gene set enrichment (CAGE) GO methodology, in which 3,875 curated gene sets of the GSEA database were correlated to TEP profiles (see Experimental Procedures), demonstrated significant correlation of TEP mRNA profiles with cancer tissue signatures, histone deacetylases regulation, and platelets (Table 2). The levels of 20 non-protein coding RNAs were altered in TEPs as compared to platelets from healthy individuals and these show a tumor type-associated RNA profile (Figure S1C).

Next, we determined the diagnostic accuracy of TEP-based pan-cancer classification in the training cohort (n = 175), employing a leave-one-out cross-validation support vector machine algorithm (SVM/LOOCV, see Experimental Procedures; Figures S1D and S1E), previously used to classify primary and metastatic tumor tissues (Ramaswamy et al., 2001, Su et al., 2001, Vapnik, 1998, Yeang et al., 2001). Briefly, the SVM algorithm (blindly) classifies each individual sample as cancer or healthy by comparison to all other samples (175 − 1) and was performed 175 times to classify and cross validate all individuals samples. The algorithms we developed use a limited number of different spliced RNAs for sample classification. To determine the specific input gene lists for the classifying algorithms we performed ANOVA testing for differences (as implemented in the R-package edgeR), yielding classifier-specific gene lists (Table S4). For the specific algorithm of the pan-cancer TEP-based classifier test we selected 1,072 RNAs (Table S4) for the n = 175 training cohort, yielding a sensitivity of 96%, a specificity of 92%, and an accuracy of 95% (Figure 1F). Subsequent validation using a separate validation cohort (n = 108), not involved in input gene list selection and training of the algorithm, yielded a sensitivity of 97%, a specificity of 94%, and an accuracy of 96% (Figure 1G), with an area under the curve (AUC) of 0.986 to detect cancer (Figure 1H) and high predictive strength (Figure 1I). In contrast, random classifiers, as determined by multiple rounds of randomly shuffling class labels (permutation) during the SVM training process (see Experimental Procedures), had no predictive power (mean overall accuracy: 78%, SD ± 0.3%, p < 0.01), thereby showing, albeit an unbalanced representation of both groups in the study cohort, specificity of our procedure. A total of 100 times random class-proportional subsampling of the entire dataset in a training and validation set (ratio 60:40) yielded similar accuracy rates (mean overall accuracy: 96%, SD: ± 2%), confirming reproducible classification accuracy in this dataset. Of note, all 39 patients with localized tumors and 33 of the 39 patients with primary tumors in the CNS were correctly classified as cancer patients (Figure 1I). Visualization of 22 genes previously identified at differential RNA levels in platelets of patients with various non-cancerous diseases (Gnatenko et al., 2010, Healy et al., 2006, Lood et al., 2010, Raghavachari et al., 2007), revealed mixed levels in our TEP dataset (Figure S1F), suggesting that the platelet RNA repertoire in patients with non-cancerous disease is distinct from patients with cancer.

### Tumor-Specific Educational Program of Blood Platelets Allows for Multiclass Cancer Diagnostics

**Figure 2:**
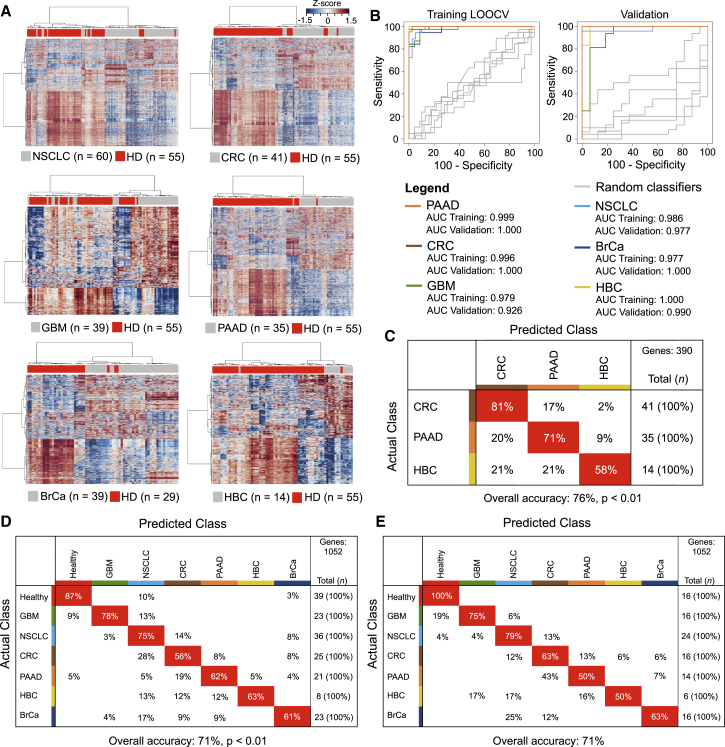
Tumor-Educated Platelet mRNA Profiles for Multiclass Cancer Diagnostics (A) Heatmaps of unsupervised clustering of platelet mRNA profiles of healthy donors (HD; n = 55) (red) and patients with non-small cell lung cancer (NSCLC; n = 60), colorectal cancer (CRC; n = 41), glioblastoma (GBM; n = 39), pancreatic cancer (PAAD, n = 35), breast cancer (BrCa; n = 39; female HD; n = 29), and hepatobiliary cancer (HBC; n = 14). (B) ROC-curve of SVM diagnostics of healthy donors and individual tumor classes in both training (left) and validation (right) cohort. Random classifiers, indicating the classification accuracies obtained by chance, are shown in gray. (C) Confusion matrix of multiclass SVM/LOOCV diagnostics of patients with CRC, PAAD, and HBC. Indicated are detection rates as compared to the actual classes in percentages. (D) Confusion matrix of multiclass SVM/LOOCV diagnostics of the training cohort consisting of healthy donors (healthy) and patients with GBM, NSCLC, PAAD, CRC, BrCa, and HBC. Indicated are detection rates as compared to the actual classes in percentages. (E) Confusion matrix of multiclass SVM algorithm in a validation cohort (n = 108). Indicated are sample numbers and detection rates in percentages. Genes, number of mRNAs included in training of the SVM algorithm. See also Figure S2 and Tables S4, S5, and S6.

In addition to the pan-cancer diagnosis, the TEP mRNA profiles also distinguished healthy donors and patients with specific types of cancer, as demonstrated by the unsupervised hierarchical clustering of differential platelet mRNA levels of healthy donors and all six individual tumor types, i.e., NSCLC, CRC, GBM, PAAD, BrCa, and HBC (Figures 2A, all p < 0.0001, Fisher’s exact test, and S2A; Table S5), and this resulted in tumor-specific gene lists that were used as input for training and validation of the tumor-specific algorithms (Table S4). For the unsupervised clustering of the all-female group of BrCa patients, male healthy donors were excluded to avoid sample bias due to gender-specific platelet mRNA profiles (Figure S2B). SVM-based classification of all individual tumor classes with healthy donors resulted in clear distinction of both groups in both the training and validation cohort, with high sensitivity and specificity, and 38/39 (97%) cancer patients with localized disease were classified correctly (Figures 2B and S2C). CAGE GO analysis showed that biological processes differed between TEPs of individual tumor types, suggestive of tumor-specific “educational” programs (Table S6). We did not detect sufficient differences in mRNA levels to discriminate patients with non-metastasized from patients with metastasized tumors, suggesting that the altered platelet profile is predominantly influenced by the molecular tumor type and, to a lesser extent, by tumor progression and metastases.

We next determined whether we could discriminate three different types of adenocarcinomas in the gastro-intestinal tract by analysis of the TEP-profiles, i.e., CRC, PAAD, and HBC. We developed a CRC/PAAD/HBC algorithm that correctly classified the mixed TEP samples (n = 90) with an overall accuracy of 76% (mean overall accuracy random classifiers: 42%, SD: ± 5%, p < 0.01, Figure 2C). In order to determine whether the TEP mRNA profiles allowed for multiclass cancer diagnosis across all tumor types and healthy donors, we extended the SVM/LOOCV classification test using a combination of algorithms that classified each individual sample of the training cohort (n = 175) as healthy donor or one of six tumor types (Figures S2D and S2E). The results of the multiclass cancer diagnostics test resulted in an average accuracy of 71% (mean overall accuracy random classifiers: 19%, SD: ± 2%, p < 0.01, Figure 2D), demonstrating significant multiclass cancer discriminative power in the platelet mRNA profiles. The classification capacity of the multiclass SVM-based classifier was confirmed in the validation cohort of 108 samples, with an overall accuracy of 71% (Figure 2E). An overall accuracy of 71% might not be sufficient for introduction into cancer diagnostics. However, of the initially misclassified samples according to the SVM algorithms choice with strongest classification strength the second ranked classification was correct in 60% of the cases. This yields an overall accuracy using the combined first and second ranked classifications of 89%. The low validation score of HBC samples can be attributed to the relative low number of samples and possibly to the heterogenic nature of this group of cancers (hepatocellular cancers and cholangiocarcinomas).

### Companion Diagnostics Tumor Tissue Biomarkers Are Reflected by Surrogate TEP mRNA Onco-signatures

**Figure 3:**
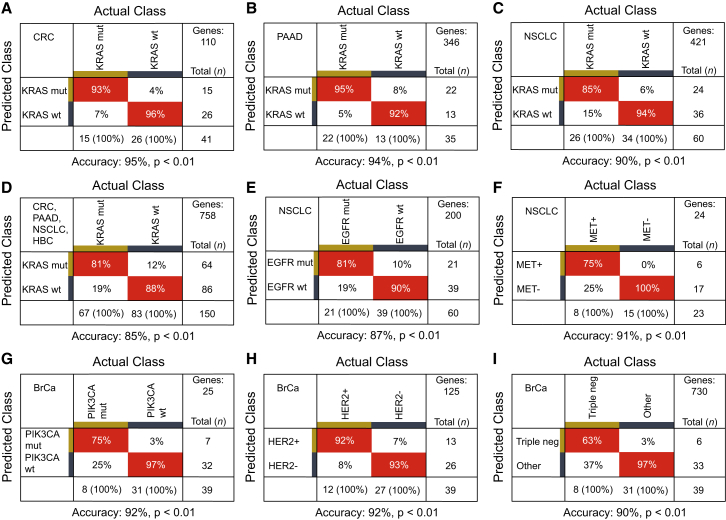
Tumor-Educated Platelet mRNA Profiles for Molecular Pathway Diagnostics Cross tables of SVM/LOOCV diagnostics with the molecular markers *KRAS* in (A) CRC, (B) PAAD, and (C) NSCLC patients, (D) *KRAS* in the combined cohort of patients with either CRC, PAAD, NSCLC, or HBC, (E) *EGFR* and (F) MET in NSCLC patients, (G) *PIK3CA* mutations, (H) *HER2*-amplification, and (I) triple negative status in BrCa patients. Genes, number of mRNAs included in training of the SVM algorithm. See also Tables S4 and S7.

Blood provides a promising biosource for the detection of companion diagnostics biomarkers for therapy selection (Bettegowda et al., 2014, Crowley et al., 2013, Papadopoulos et al., 2006). We selected platelet samples of patients with distinct therapy-guiding markers confirmed in matching tumor tissue. Although the platelet mRNA profiles contained undetectable or low levels of these mutant biomarkers, the TEP mRNA profiles did allow to distinguish patients with *KRAS* mutant tumors from *KRAS* wild-type tumors in PAAD, CRC, NSCLC, and HBC patients, and *EGFR* mutant tumors in NSCLC patients, using algorithms specifically trained on biomarker-specific input gene lists (all p < 0.01 versus random classifiers, Figures 3A–3E; Table S4). Even though the number of samples analyzed is relatively low and the risk of algorithm overfitting needs to be taken into account, the TEP profiles distinguished patients with *HER2*-amplified, *PIK3CA* mutant or triple-negative BrCa, and NSCLC patients with MET overexpression (all p < 0.01 versus random classifiers, Figures 3F–3I).

We subsequently compared the diagnostic accuracy of the TEP mRNA classification method with a targeted *KRAS* (exon 12 and 13) and *EGFR* (exon 20 and 21) amplicon deep sequencing strategy (∼5,000× coverage) on the Illumina Miseq platform using prospectively collected blood samples of patients with localized or metastasized cancer. This method did allow for the detection of individual mutant *KRAS* and *EGFR* sequences in both plasma DNA and platelet RNA (Table S7), indicating sequestration and potential education capacity of mutant, tumor-derived RNA biomarkers in TEPs. Mutant *KRAS* was detected in 62% and 39%, respectively, of plasma DNA (n = 103, kappa statistics = 0.370, p < 0.05) and platelet RNA (n = 144, kappa statistics = 0.213, p < 0.05) of patients with a *KRAS* mutation in primary tumor tissue. The sensitivity of the plasma DNA tests was relatively poor as reported by others (Bettegowda et al., 2014, Thierry et al., 2014), which may partly be attributed to the loss of plasma DNA quality due to relatively long blood sample storage (EDTA blood samples were stored up to 48 hr at room temperature before plasma isolation). To discriminate *KRAS* mutant from wild-type tumors in blood, the TEP mRNA profiles provided superior concordance with tissue molecular status (kappa statistics = 0.795–0.895, p < 0.05) compared to *KRAS* amplicon sequencing analysis of both plasma DNA and platelet RNA (Table S7). Thus, TEP mRNA profiles can harness potential blood-based surrogate onco-signatures for tumor tissue biomarkers that enable cancer patient stratification and therapy selection.

### TEP-Profiles Provide an All-in-One Biosource for Blood-Based Liquid Biopsies in Patients with Cancer

**Figure 4:**
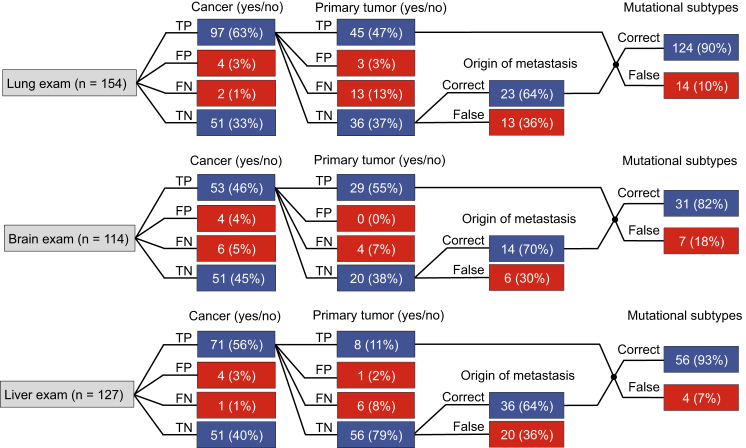
Organ-Focused TEP-Based Cancer Diagnostics SVM/LOOCV diagnostics of healthy donors (n = 55) and patients with primary or metastatic tumor burden in the lung (n = 99; totaling 154 tests), brain (n = 62; totaling 114 tests), or liver (n = 72; totaling 127 tests), to determine the presence or absence of cancer, with cancer subclassified as primary or metastatic tumor, in case of metastases the identified organ of origin, and the correctly identified molecular markers. Of note, at the exam level of mutational subtypes some samples were included in multiple classifiers (i.e., *KRAS*, *EGFR*, *PIK3CA*, *HER2*-amplification, MET-overexpression, or triple negative status), explaining the higher number in mutational tests than the total number of included samples. TP, true positive; FP, false positive; FN, false negative; TN, true negative. Indicated are sample numbers and detection rates in percentages.

Unequivocal discrimination of primary versus metastatic nature of a tumor may be difficult and hamper adequate therapy selection. Since the TEP profiles closely resemble the different tumor types as determined by their organ of origin—regardless of systemic dissemination—this potentially allows for organ-specific cancer diagnostics. Hence we selected all healthy donors and all patients with primary or metastatic tumor burden in the lung (n = 154), brain (n = 114), or liver (n = 127). We performed “organ exams” and instructed the SVM/LOOCV algorithm to determine for lung, brain, and liver the presence or absence of cancer (96%, 91%, and 96% accuracy, respectively), with cancer subclassified as primary or metastatic tumor (84%, 93%, and 90% accuracy, respectively) and in case of metastases to identify the potential organ of origin (64%, 70%, and 64% accuracy, respectively). The platelet mRNA profiles enabled assignment of the cancer to the different organs with high accuracy (Figure 4). In addition, using the same TEP mRNA profiles we were able to again indicate the biomarker status of the tumor tissues (90%, 82%, and 93% accuracy, respectively) (Figure 4).

## Discussion

The use of blood-based liquid biopsies to detect, diagnose, and monitor cancer may enable earlier diagnosis of cancer, lower costs by tailoring molecular targeted treatments, improve convenience for cancer patients, and ultimately supplements clinical oncological decision-making. Current blood-based biosources under evaluation demonstrate suboptimal sensitivity for cancer diagnostics, in particular in patients with localized disease. So far, none of the current blood-based biosources, including plasma DNA, exosomes, and CTCs, have been employed for multiclass cancer diagnostics (Alix-Panabières and Pantel, 2014, Bettegowda et al., 2014, Skog et al., 2008), hampering its implementation for early cancer detection. Here, we report that molecular interrogation of blood platelet mRNA can offer valuable diagnostics information for all cancer patients analyzed—spanning six different tumor types. Our results suggest that platelets may be employable as an all-in-one biosource to broadly scan for molecular traces of cancer in general and provide a strong indication on tumor type and molecular subclass. This includes patients with localized disease possibly allowing for targeted diagnostic confirmation using routine clinical diagnostics for each particular tumor type.

Since the discovery of circulating tumor material in blood of patients with cancer (Leon et al., 1977) and the recognition of the clinical utility of blood-based liquid biopsies, a wealth of studies has assessed the use of blood for cancer diagnostics, prognostication and treatment monitoring (Alix-Panabières et al., 2012, Bidard et al., 2014, Crowley et al., 2013, Haber and Velculescu, 2014). By development of highly sensitive targeted detection methods, such as targeted deep sequencing (Newman et al., 2014), droplet digital PCR (Bettegowda et al., 2014), and allele-specific PCR (Maheswaran et al., 2008, Thierry et al., 2014), the utility and applicability of liquid biopsies for clinical implementation has accelerated. These advances previously allowed for a pan-cancer comparison of various biosources and revealed that in >75% of cancers, including advanced stage pancreas, colorectal, breast, and ovarian cancer, cell-free DNA is detectable although detection rates are dependent on the grade of the tumor and depth of analysis (Bettegowda et al., 2014). Here, we show that the platelet RNA profiles are affected in nearly all cancer patients, regardless of the type of tumor, although the abundance of tumor-associated RNAs seems variable among cancer patients. In addition, surrogate RNA onco-signatures of tissue biomarkers, also in 88% of localized KRAS mutant cancer patients as measured by the tumor-specific and pan-cancer SVM/LOOCV procedures, are readily available from a minute amount (100–500 pg) of platelet RNA. As whole blood can be stored up to 48 hr on room temperature prior to isolation of the platelet pellet, while maintaining high-quality RNA and the dominant cancer RNA signatures, TEPs can be more readily implemented in daily clinical laboratory practice and could potentially be shipped prior to further blood sample processing.

Blood platelets are widely involved in tumor growth and cancer progression (Gay and Felding-Habermann, 2011). Platelets sequester solubilized tumor-associated proteins (Klement et al., 2009) and spliced and unspliced mRNAs (Calverley et al., 2010, Nilsson et al., 2011), whereas platelets do also directly interact with tumor cells (Labelle et al., 2011), neutrophils (Sreeramkumar et al., 2014), circulating NK-cells (Palumbo et al., 2005, Placke et al., 2012), and circulating tumor cells (Ting et al., 2014, Yu et al., 2013). Interestingly, in vivo experiments have revealed breast cancer-mediated systemic instigation by supplying circulating platelets with pro-inflammatory and pro-angiogenic proteins, supporting outgrowth of dormant metastatic foci (Kuznetsov et al., 2012). Using a gene ontology methodology, CAGE, we correlated TEP-cancer signatures with publicly available curated datasets. Indeed, we identified widespread correlations with cancer tissues, hypoxia, platelet-signatures, and cytoskeleton, possibly reflecting the “alert” and pro-tumorigenic state of TEPs. We observed strong negative correlations with RNAs implicated in RNA translation, T cell immunity, and interleukin-signaling, implying diminished needs of TEPs for RNAs involved in these biological processes or orchestrated translation of these RNAs to proteins (Denis et al., 2005). We observed that the tumor-specific educational programs in TEPs are predominantly influenced by tumor type and, to a lesser extent, by tumor progression and metastases. Although we were not able to measure significant differences between non-metastasized and metastasized tumors, we do not exclude that the use of larger sample sets could allow for the generation of SVM algorithms that do have the power to discriminate between certain stages of cancer, including those with in situ carcinomas and even pre-malignant lesions. In addition, different molecular tumor subtypes (e.g., *HER2*-amplified versus wild-type BrCa) result in different effects on the platelet profiles, possibly caused by different “educational” stimuli generated by the different molecular tumor subtypes (Koboldt et al., 2012). Altogether, the RNA content of platelets in patients with cancer is dependent on the transcriptional state of the bone-marrow megakaryocyte (Calverley et al., 2010, McAllister and Weinberg, 2014), complemented by sequestration of spliced RNA (Nilsson et al., 2011), release of RNA (Clancy and Freedman, 2014, Kirschbaum et al., 2015, Rak and Guha, 2012, Risitano et al., 2012), and possibly queue-specific pre-mRNA splicing during platelet circulation. Partial or complete normalization of the platelet profiles following successful treatment of the tumor would enable TEP-based disease recurrence monitoring, requiring the analysis of follow-up platelet samples. Future studies will be required to address the tumor-specific “educated” profiles on both an (small non-coding) RNA (Laffont et al., 2013, Landry et al., 2009, Leidinger et al., 2014, Lu et al., 2005) and protein (Burkhart et al., 2014, Geiger et al., 2013, Klement et al., 2009) level and determine the ability of gene ontology, blood-based cancer classification.

In conclusion, we provide robust evidence for the clinical relevance of blood platelets for liquid biopsy-based molecular diagnostics in patients with several types of cancer. Further validation is warranted to determine the potential of surrogate TEP profiles for blood-based companion diagnostics, therapy selection, longitudinal monitoring, and disease recurrence monitoring. In addition, we expect the self-learning algorithms to further improve by including significantly more samples. For this approach, isolation of the platelet fraction from whole blood should be performed within 48 hr after blood withdrawal, the platelet fraction can subsequently be frozen for cancer diagnosis. Also, future studies should address causes and anticipated risks of outlier samples identified in this study, such as healthy donors classified as cancer patients. Systemic factors such as chronic or transient inflammatory diseases, or cardiovascular events and other non-cancerous diseases may also influence the platelet mRNA profile and require evaluation in follow-up studies, possibly also including individuals predisposed for cancer.

## Experimental Procedures

### Sample Collection and Study Oversight

Blood was drawn from all patients and healthy donors at the VU University Medical Center, Amsterdam, the Netherlands, or the Massachusetts General Hospital (MGH), Boston, in 6 ml purple-cap BD Vacutainers containing the anti-coagulant EDTA. To minimize effects of long-term storage of platelets at room temperature and loss of platelet RNA quality and quantity, samples were processed within 48 hr after blood collection. Blood samples of patients were collected pre-operatively (GBM) or during follow-up in the outpatient clinic (CRC, NSCLC, PAAD, BrCa, HBC). Nine cancer patient samples included were follow-up samples of the same patient collected within months of the first blood collection (five samples in NSCLC, two samples in PAAD, and one sample in BrCa and HBC). Localized disease cancer patients were defined as cancer patients without known metastasis from the primary tumor to distant organ(s), as noticed by the physician or additional imaging and/or pathological tests. Patients with glioblastoma, a tumor that metastasizes rarely, were regarded as late-stage (high-grade) cancers. Samples for both training and validation cohort were collected and processed similarly and simultaneously. Tumor tissues of patients were analyzed for the presence of genetic alterations by tissue DNA sequencing, including next-generation sequencing SNaPShot, assessing 39 genes over 152 exons with an average sequencing coverage of >500, including *KRAS*, *EGFR*, and *PIK3CA* (Dias-Santagata et al., 2010). Assessment of MET overexpression in non-small cell lung cancer FFPE slides was performed by immunohistochemistry (anti-Total cMET SP44 Rabit monoclonal antibody (mAb), Ventana, or the A2H2-3 anti-human MET mAb (Gruver et al., 2014)). The estrogen and progesterone status of BrCa tumor tissues and the *HER2* amplification of BrCa tumor tissue were determined using immunohistochemistry and fluorescent in situ hybridization, respectively, and scored according to the routine clinical diagnostics protocol at the MGH, Boston. Healthy donors were at the moment of blood collection, or previously, not diagnosed with cancer. This study was conducted in accordance with the principles of the Declaration of Helsinki. Approval was obtained from the institutional review board and the ethics committee at each hospital, and informed consent was obtained from all subjects. Clinical follow-up of healthy donors is not available due to anonymization of these samples according to the ethical rules of the hospitals.

### Support Vector Machine Classifier

For binary (pan-cancer) and multiclass sample classification, a support vector machine (SVM) algorithm was used implemented by the e1071 R-package. In principal, the SVM algorithm determines the location of all samples in a high-dimensional space, of which each axis represents a transcript included and the sample expression level of a particular transcript determines the location on the axis. During the training process, the SVM algorithm draws a hyperplane best separating two classes, based on the distance of the closest sample of each class to the hyperplane. The different sample classes have to be positioned at each side of the hyperplane. Following, a test sample with masked class identity is positioned in the high-dimensional space and its class is “predicted” by the distance of the particular sample to the constructed hyperplanes. For the multiclass SVM classification algorithm, a One-Versus-One (OVO) approach was used. Here, each class is compared to all other individual classes and thus the SVM algorithm defines multiple hyperplanes. To cross validate the algorithm for all samples in the training cohort, the SVM algorithm was trained by all samples in the training cohort minus one, while the remaining sample was used for (blind) classification. This process was repeated for all samples until each sample was predicted once (leave-one-out cross-validation [LOOCV] procedure). The percentage of correct predictions was reported as the classifier’s accuracy. To assess the predictive value of the SVM algorithm on an independent dataset, which is not previously involved in the SVM training process and thus entirely new for the algorithm, the algorithm was trained on the training dataset, all SVM parameters were fixed, and the samples belonging to the validation cohort were predicted. In addition, an iterative (100×) process was performed in which samples of the dataset were randomly subsampled in a training and validation set (ratio training:validation = 60:40 (all cancer classes) or 70:30 (healthy individuals), per sample class samples were subsampled in this ratio according the total size of the individual classes (class-proportional, stratified subsampling)) and mean accuracy of all individual classifications was reported. Internal performance of the SVM algorithm could be improved by enabling the SVM tuning function, which implies optimal determination of parameters of the SVM algorithm (gamma, cost) by randomly subsampling the dataset used for training (“internal cross-validation”) of the algorithm. Prior to construction of the SVM algorithm, transcripts with low expression (<5 reads in all samples) were excluded and read counts were normalized as described in the Supplemental Experimental Procedures (differential expression of transcripts). For each individual prediction, feature selection (identification of transcripts with notable influence on the predictive performance) was performed by ANOVA testing for differences, yielding classifier-specific input gene lists (Table S4). mRNAs with a LogCPM >3 and a p value corrected for multiple hypothesis testing (FDR) of <0.95 (pan-cancer *KRAS*), <0.90 (CRC, PAAD, and NSCLC *KRAS* and *HER2*-amplified BrCa), <0.80 (*PIK3CA* BrCa), <0.70 (NSCLC *EGFR*), <0.50 (triple negative-status BrCa), <0.30 (MET-overexpression NSCLC), <0.10 (CRC/PAAD/HBC), <0.0001 (multiclass tumor type and individual tumor class-healthy), and <0.00005 (pan-cancer/healthy-cancer) were included. Internal SVM tuning was enabled to improve predictive performance. All individual tumor class versus healthy donors and molecular pathway SVMs algorithms were tuned by a (default) 10-fold internal cross-validation. The pan-cancer/healthy-cancer, multiclass tumor type, and the gastro-intestinal CRC/PAAD/HBC SVM algorithms were tuned by a 2-fold internal cross-validation. The training cohort of the pan-cancer and multiclass tumor type, the individual tumor classes versus healthy donor tests, the gastro-intestinal CRC/PAAD/HBC test, and all molecular pathway tests were analyzed using a LOOCV approach. To increase classification specificity in the multiclass tumor type test, additional binary and multiclass classifiers algorithms were developed, namely the pan-cancer test (Figures 1F and 1G), HBC-CRC, HBC-PAAD, BrCa-CRC, BrCa-CRC-NSCLC, and BrCa-HD-GBM-NSCLC tests, evaluated in both the training and validation cohort separately, which were applied sequentially to the multiclass tumor type test. Samples predicted as either condition of the supplemental classifier were all re-evaluated using the filter. The latter tumor class classification was regarded as the follow-up classification. In addition, samples predicted as the all-female breast cancer class, but of male origin as determined by the gender-specific RNAs (Figure S2B), and samples predicted as healthy, while in the pan-cancer test predicted as cancer, were automatically assigned to the class with second predictive strength, as supplemented by the SVM output. To determine the accuracy rates of the classifiers that can be obtained by chance, class labels of the samples used by the SVM algorithm for training were randomly permutated (“random classifiers”). This process was performed for 100 LOOCV classification procedures. P values were determined by counting the overall random classifier LOOCV-classification accuracies that yielded similar or higher total accuracy rates compared to the observed total accuracy rate. The predictive strength was also used as input to generate a receiver operating curve (ROC) as implemented in the R-package pROC (version 1.7.3). Organ exams were calculated based on the compiled results of the SVM/LOOCV of the training cohort and subsequent prediction of the validation cohort, spanning in total 283 samples. The pan-cancer binary SVM, the multiclass SVM, and all molecular pathway SVM algorithms were processed individually. Samples included for each organ exam (all healthy donors, all samples with primary tumor in a particular organ, and all samples with known metastases to the particular organ) were selected. Only samples with correct predictions at a particular level of the organ exam were passed to the next level for evaluation. Counts of correct and false predictions in the “mutational subtypes”-stage were determined from all individual molecular pathway SVM algorithms in which the selected samples were included.

### Correlative Analysis of Gene Set Enrichment Analysis

Correlative Analyses of Gene Set Enrichment (CAGE) analysis was performed in the online platform R2 (R2.amc.nl). To enable analyses of RNA-sequencing read counts in a micro-array-based statistical platform, counts per million normalized read counts were voom-transformed, using sequencing batch and sample group as variables, and uploaded in the R2-environment. Highly correlating mRNAs (FDR < 0.01) of a tumor type or all tumor classes combined (pan-cancer) compared to all other classes was used to generate a class-specific gene signature. These individual signatures were subsequently correlated with 3,875 curated gene sets as provided by the Broad Institute (http://www.broadinstitute.org/gsea). Top 25 ranking correlations were manually annotated by two independent researchers (M.G.B. and B.A.W.) and shared annotated terms were after agreement of both researchers reported.

## Author Contributions

M.G.B., B.A.T., P.W., and T.W. designed the study and wrote the manuscript. E.F.S., D.P.N., H.M.V., J.C.R., and B.A.T. provided clinical samples. M.G.B., N.S., J.T., F.R., P.S., J.D., B.Y., H.V., and E.P. performed sample processing for mRNA-seq. R.J.A.N., P.S., H.V., E.P., and T.W. designed and performed amplicon sequencing assays. M.G.B., N.S., I.K., J.D., B.A.W., J.K., N.A., E.P., and T.W. performed data analyses and interpretation. All authors provided critical comments on the manuscript.

## Conflicts of Interest

P.S, H.V., E.P., R.J.A.N., and T.W. are employees of thromboDx BV. R.J.A.N. and T.W. are shareholders and founders of thromboDx BV.

## Figures and Tables

**Table 1:** Summary of Patient Characteristics

Patient Group	Total (n)		Gender M (%)^a^		Age (SD)^b^		Metastasis (%)		Mutation	Presence (%)	
Training	Validation	Training	Validation	Training	Validation	Training	Validation	Training	Validation
HD	39	16	21 (54)	6 (38)	41 (13)	38 (16)	–	–	–	–	–
GBM	23	16	18 (78)	10 (63)	59 (16)	62 (14)	0 (0)	0 (0)	–	–	–
NSCLC	36	24	14 (39)	14 (58)	60 (11)	59 (12)	33 (92)	23 (96)	*KRAS*	15 (42)	11 (46)
									*EGFR*	14 (39)	7 (29)
									MET-overexpression	5 (14)	3 (13)
CRC	25	16	13 (52)	9 (56)	59 (13)	63 (16)	20 (80)	15 (94)	*KRAS*	7 (28)	8 (50)
PAAD	21	14	12 (57)	7 (50)	66 (9)	66 (10)	15 (71)	9 (64)	*KRAS*	13 (62)	9 (64)
BrCa	23	16	0 (0)	0 (0)	59 (11)	59 (11)	16 (70)	9 (56)	*HER2*^*+*^	7 (30)	5 (31)
									*PIK3CA*	6 (26)	2 (13)
									triple negative	5 (22)	3 (19)
HBC	8	6	6 (75)	2 (33)	68 (13)	62 (16)	6 (75)	4 (67)	*KRAS*	3 (38)	1 (17)

HD, healthy donors; GBM, glioblastoma; NSCLC, non-small cell lung cancer; CRC, colorectal cancer; PAAD, pancreatic cancer; BrCa, breast cancer; HBC, hepatobiliary cancer. See also Table S1.

a Indicated are number of male individuals.

b Indicated is mean age in years.

**Table 2:** Pan-Cancer CAGE Gene Ontology

	Top 25 GO Correlations		
#	Lowest^a^	Highest^a^
**Down**			
Translation	10	−0.865	−0.890
Immune, T cell	5	−0.853	−0.883
Cancer-associated	2	−0.875	−0.887
Viral replication	2	−0.875	−0.878
IL-signaling	2	−0.869	−0.874
RNA processing	1	−0.886	
Ago2-Dicer-silencing	1	−0.882	
Protein metabolism	1	−0.879	
Receptor processing	1	−0.869	

**Up**			
Cancer-associated	6	−0.783	−0.906
Infection	3	−0.798	−0.853
HDAC	3	−0.795	−0.852
Platelet	3	−0.837	−0.906
Cytoskeleton	2	−0.801	−0.886
Hypoxia	2	−0.763	−0.937
Protease	1	−0.854	
Immunodeficiency	1	−0.812	
Differentiation	1	−0.810	
Immune differentiation	1	−0.801	
Methylation	1	−0.778	
Metabolism	1	−0.768	

Top-ranking correlations of platelet-mRNA profiles with 3,875 Broad Institute curated gene sets. CAGE, Correlative Analysis of Gene Set Enrichment; GO, gene ontology; #, number of hits per annotation; IL, interleukin; HDAC, histone deacetylase.

a Indicated are lowest and highest correlations per annotation.

## Accession Numbers

The accession number for the raw sequencing data reported in this paper is GEO: GSE68086.

## Supplemental Information

Document S1. Supplemental Experimental Procedures and Figures S1 and S2

Table S1. Patient Characteristics, Related to Table 1

Table S2. Differentially Expressed mRNAs between Healthy Donor Platelets and TEPs, Related to Figure 1

Table S3. DAVID Gene Ontology of Significantly Enriched and Decreased mRNAs in TEPs, Related to Figure 1

Table S4. SVM Classifier-Specific Gene Lists, Related to Figure 1

Table S5. Differentially Expressed mRNAs between Healthy Donor Platelets and TEPs of Individual Tumor Types, Related to Figure 2

Table S6. Correlative Analysis of Gene Set Enrichment Gene Ontology of All Tumor Classes, Related to Figure 2

Table S7. Comparison of Plasma DNA and TEP mRNA Amplicon Sequencing and TEP mRNA Sequencing, Related to Figure 3

Document S2. Article plus Supplemental Information
