# Supplementary material for: RNA-Seq of Tumor-Educated Platelets Enables Blood-Based Pan-Cancer, Multiclass, and Molecular Pathway Cancer Diagnostics
Source: Cancer Cell. 2015 Nov 9;28(5):666–76. doi: 10.1016/j.ccell.2015.09.018 (PMC4644263; doi:10.1016/j.ccell.2015.09.018)
Supplement: Document S1. Supplemental Experimental Procedures and Figures S1 and S2 [file mmc1.pdf]

Cancer Cell, Volume *28*

Supplemental Information

RNA-Seq of Tumor-Educated Platelets Enables Blood-Based Pan-Cancer, Multiclass, and Molecular Pathway Cancer Diagnostics

Myron G. Best, Nik Sol, Irsan Kooi, Jihane Tannous, Bart A. Westerman, François Rustenburg, Pepijn Schellen, Heleen Verschueren, Edward Post, Jan Koster, Bauke Ylstra, Najim Ameziane, Josephine Dorsman, Egbert F. Smit, Henk M. Verheul, David P. Noske, Jaap C. Reijneveld, R. Jonas A. Nilsson, Bakhos A. Tannous, Pieter Wesseling, and Thomas Wurdinger

SUPPLEMENTAL DATA

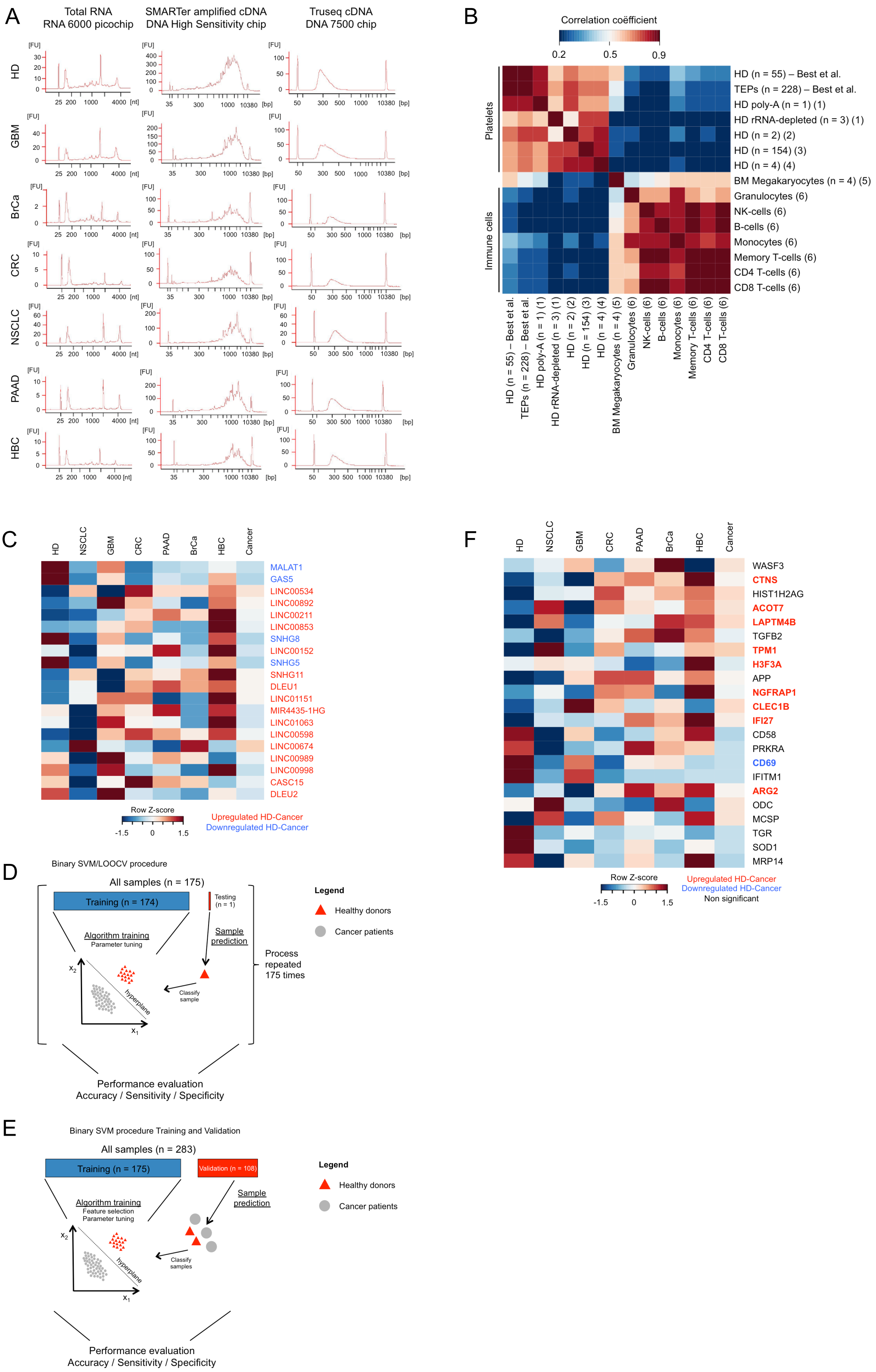

Figure S1. Additional data and schematic figures for clarification of the SVM process, related to Figure 1.

(**A**) Bioanalyzer graphs shown for each tumor class and healthy donors after total RNA isolation, SMARTer cDNA amplification, and Truseq library preparation. X-axis represents length of detected molecules, y-axis represents measured fluorescent signal. (**B**) Pearson correlation (color bar) matrix of publicly available dataset (columns and rows), consisting of four datasets with platelets from healthy donors, one dataset of megakaryocytes, and one dataset containing seven different circulating immune cells, and sequenced platelets and TEPs. If applicable, the number of individuals per datasets was noted. The different platelet datasets were highly correlated, whereas no correlation was observed with circulating immune cells. Publicly available datasets were obtained from: 1. Kissopoulou et al., 2. Rowley et al., 3. Simon et al., 4. Bray et al., 5. Chen et al., and 6. Hrdlickova et al. HD = healthy donors, TEP = tumor-educated platelets, BM = bone-marrow. (**C**) Heatmap of differential levels (FDR < 0.001) of 20 non-protein coding RNAs sorted by FDR value as determined by the analysis of the differential levels of RNAs between healthy and cancer platelets. Enriched RNAs are shown in red, decreased RNAs are shown in blue. (**D**) According to a LOOCV procedure, all samples (n = 175) are subdivided in 174 samples for training and one sample for testing. Samples for training are positioned in a high-dimensional space in which each axis represents a gene included in the SVM algorithm (denoted as x_1, x_2, etc.). A hyperplane best separating both groups is identified by the SVM algorithm. SVM performance is improved by feature selection and internal parameter tuning. Following, the test sample is classified and this process is repeated for each sample. For evaluation, samples with low predictive strength were excluded and SVM performance was reported (accuracy, sensitivity, specificity). (**E**) Evaluation of SVM performance in validation cohort. Training of SVM algorithm is performed using the training cohort (n = 175) as a reference. Subsequently, 108 samples (validation cohort) not involved in training of the algorithm are classified. (**F**) Heatmap shows the average group counts per million levels of 22 RNA markers in TEPs and platelets of healthy donors. These markers were previously identified in platelets of patients with essential and reactive thrombocytosis (Gnatenko et al. Blood 2010), systemic lupus erythematosus (Lood et al. Blood 2010), sickle cell disease (Raghavachari et al. Circulation 2007), and cardiovascular disease (Healy et al. Circulation 2006). RNAs enriched in TEPs, as

determined by the differential expression analysis between platelets from healthy individuals and TEPs (see Figure 1D and E), are shown in red, decreased RNAs are shown in blue, and non-significantly altered RNAs are shown in black.

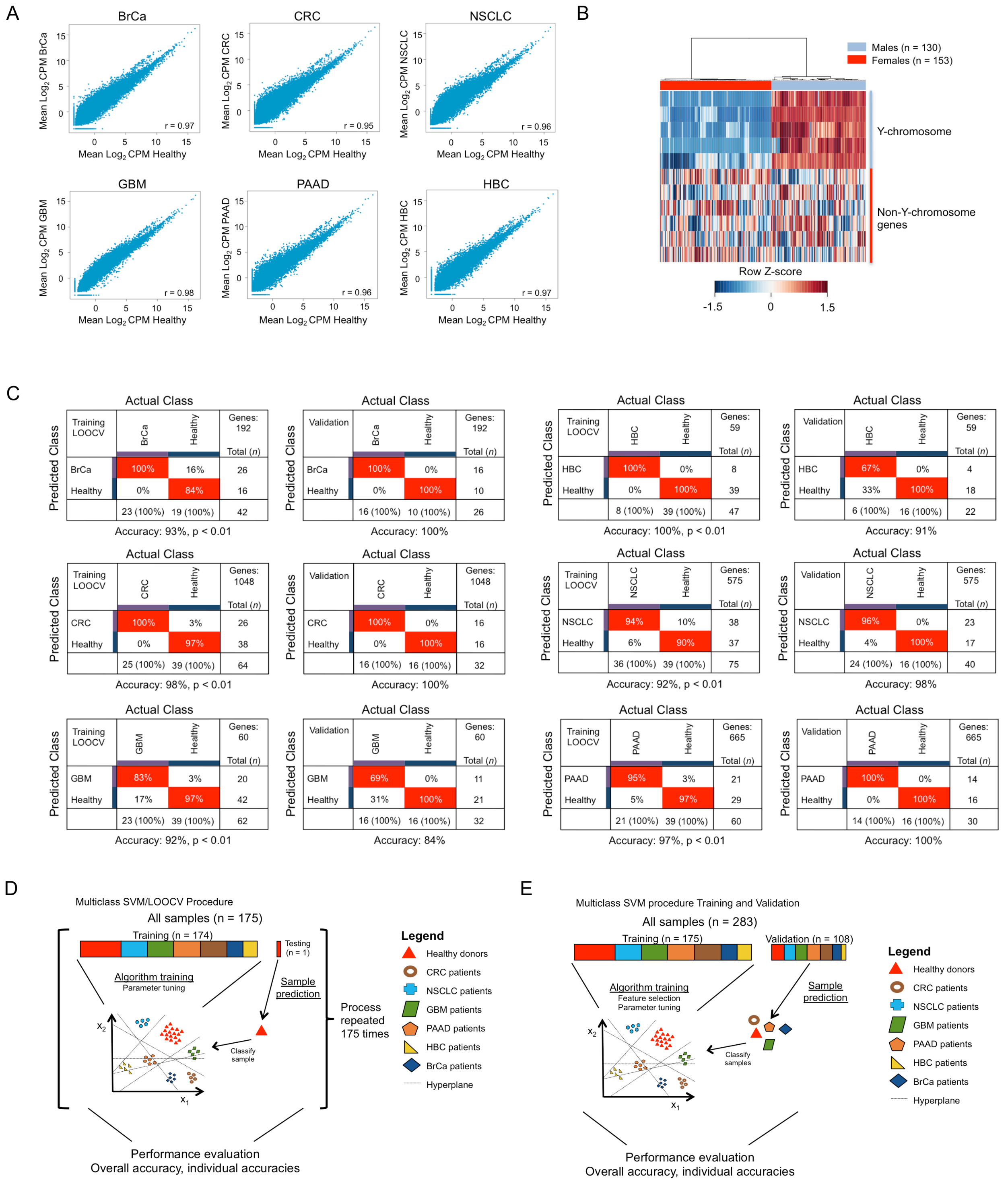
A
BrCa
CRC
NSCLC
GBM
PAAD
HBC
Mean Log2 CPM Healthy
r = 0.97
r = 0.95
r = 0.96
r = 0.98
r = 0.96
r = 0.97
B
Males (n = 130)
Females (n = 153)
Y-chromosome
Non-Y-chromosome genes
Row Z-score
-1.5
0
1.5
C
Actual Class
Predicted Class
Training LOOCV
Validation
Accuracy: 93%, p < 0.01
Accuracy: 100%
Accuracy: 100%, p < 0.01
Accuracy: 91%
Accuracy: 98%, p < 0.01
Accuracy: 100%
Accuracy: 92%, p < 0.01
Accuracy: 98%
Accuracy: 92%, p < 0.01
Accuracy: 84%
Accuracy: 97%, p < 0.01
Accuracy: 100%
D
Multiclass SVM/LOOCV Procedure
All samples (n = 175)
Training (n = 174)
Testing (n = 1)
Algorithm training
Parameter tuning
Sample prediction
Classify sample
Process repeated 175 times
Performance evaluation
Overall accuracy, individual accuracies
E
Multiclass SVM procedure Training and Validation
All samples (n = 283)
Training (n = 175)
Validation (n = 108)
Algorithm training
Feature selection
Parameter tuning
Sample prediction
Classify samples
Performance evaluation
Overall accuracy, individual accuracies
Legend
Healthy donors
CRC patients
NSCLC patients
GBM patients
PAAD patients
HBC patients
BrCa patients
Hyperplane

Figure S2. Additional data and schematic figures, related to Figure 2.

(**A**) Pearson correlations of healthy donors (x-axis) and BrCa (r = 0.97), CRC (r = 0.95), NSCLC (r = 0.96), GBM (r = 0.98), PAAD (r = 0.96), and HBC (r = 0.97) (y-axis) show high concordance between these sample classes. Per class, mean Log_2-transformed counts per million (CPM) were used. (**B**) Differential levels of mRNAs (FDR < 0.05) detected in platelets between male (blue, n = 130) and female (red, n = 153) individuals. mRNAs located on chromosomes 1-22 and X were distinguished from mRNAs encoded by the Y-chromosome. (**C**) Cross table of SVM/LOOCV diagnostics with healthy donor subjects and the tumor classes BrCa, CRC, GBM, HBC, NSCLC, and PAAD. Unique tumor-specific gene lists were determined by ANOVA analysis and used to train the different algorithms (see Table S4). For the BrCa-classifying algorithm, only female healthy donors were included. Columns show the real sample class and rows indicate the predicted class. Indicated are sample numbers and detection rates in percentages. Accuracy performance for each algorithm is indicated below the cross table. All experiments yielded a substantially higher accuracy compared to random classifiers (100 LOOCV iterations performed, p < 0.01). (**D**) For multiclass classification, seven classes (i.e. six tumor class and healthy donors) were included in the SVM procedure. First, according to a LOOCV procedure, all training samples (n = 175) are subdivided in 174 samples for training and one sample for testing. Samples for training are positioned in a high-dimensional space in which each axis represents a gene included in the SVM algorithm (denoted as x_1, x_2, etc.). Hyperplanes best separating all groups are identified by the SVM algorithm. SVM algorithm performance is improved by feature selection and internal parameter tuning. Following, the test sample is classified and this process is repeated for each sample. For evaluation, samples with low predictive strength were excluded and SVM algorithm performance was reported (overall accuracy, individual accuracies). (**E**) Evaluation of SVM performance in validation cohort. Training of SVM algorithm is performed using the training cohort (n = 175) as a reference. Subsequently, 108 samples (validation cohort) not involved in training of the algorithm are classified.

SUPPLEMENTAL EXPERIMENTAL PROCEDURES

Isolation of platelet RNA and plasma DNA and assessment of platelet purity

Platelets and plasma were isolated from whole blood collected prospectively in purple-cap BD Vacutainers containing EDTA anti-coagulant by standard centrifugation as described previously (Nilsson et al., 2011). The cells and aggregates were removed by centrifugation at room temperature for 20 min at 120g, resulting in platelet-rich plasma. The platelets were isolated from the platelet-rich plasma by centrifugation at room temperature for 20 min at 360g, after which the plasma was centrifuged at 5000 rpm for 10 min and frozen. The platelet pellet was collected in 30 µl RNAlater (Life Technologies), incubated overnight at 4°C and frozen at -80°C for further use. Plasma was stored directly at -80°C. To assess sample purity, seven freshly isolated and randomly selected platelet isolations in RNAlater were fixed in 3.7% paraformaldehyde and stained by Crystal-Violet staining (ratio 1:1). Total platelet and nucleated cell counts was determined by manual cell counting in 7 µl cell counting chambers on an light microscope by two observers (M.G.B. and T.W.) and yielded an estimated 1 to 5 nucleated cell counts per 10 million platelets, which is in concordance to observations by others (Rolf, 2005). Plasma DNA was isolated using the QiaAmp DNA Blood Mini Kit (Qiagen). The plasma DNA concentration and quality was determined using the Bioanalyzer 2100 with DNA High Sensitivity chip (Agilent). Plasma DNA was stored at -20°C for amplicon sequencing. Frozen platelets were thawed on ice and total RNA was isolated using the mirVana RNA isolation kit (Life Technologies) according to the manufacturers' protocol. Complementary purification of small RNAs was included in the isolation procedure by addition of miRNA homogenate (Life Technologies). Total RNA was dissolved in 30 µl Elution Buffer (Life Technologies) and RNA quality and quantity was measured using the RNA 6000 Picochip (Agilent).

Platelet mRNA sequencing

Platelet total RNA (Bioanalyzer RIN values > 7 and/or distinctive rRNA curves) was subjected to cDNA synthesis and amplification using the SMARTer Ultra Low RNA Kit for Illumina Sequencing v1 (Clontech, cat. nr. 634936) according to the manufacturers' protocol (Ramsköld et al., 2012). Conversion and efficient amplification of cDNA was quality controlled using the Bioanalyzer 2100 with DNA High Sensitivity chip (Agilent). Samples with detectable fragments in the 300 - 7500 bp region were selected for further processing and Covaris shearing by sonication (Covaris Inc). Sample preparation for Illumina sequencing was performed using the Truseq DNA Sample Prep Kit (Illumina, cat nr. FC-121-2001) or Truseq Nano DNA Sample Prep Kit (Illumina, cat nr. FC-121-4001). Sample quality and quantity was measured using the DNA 7500 chip or DNA High Sensitivity chip (Agilent). High-quality samples with product sizes between 300 - 500 bp were pooled in equimolar concentrations and submitted for 100 bp Single Read sequencing on the Hiseq 2500 platform (Illumina).

Processing of raw mRNA sequencing data to read count matrix

For each sample the obtained sequencing reads were cleaned by 5'-end quality trimming and clipping of the sequencing adapters by Trimmomatic (Bolger et al., 2014). Pre-alignment quality control of the cleaned sequencing reads was performed with FastQC (Andrews and others, 2010). Spliced alignment to reference genome hg19 of cleaned sequencing reads was performed with STAR (Dobin et al., 2013), guided by Ensembl gene annotation version 75. Post-alignment quality control including genebody coverage analysis was performed with RSeQC (Wang et al.,

2012). Read summarization of only reads spanning introns (intron-spanning, IS) was performed with HTseq (Anders et al., 2014), using union intersection of uniquely aligned reads with Ensembl gene annotation version 75. Both protein coding and non-coding RNAs were included during the read mapping, summarization and further downstream analyses. Genes encoded on the mitochondrial DNA and the Y-chromosome were excluded from the analyses. Samples that yielded less than $0.4x10^6$ IS reads were excluded for analyses (i.e. 5 samples out of 288 sequenced platelet samples). All subsequent statistical analyses were performed in R (version 3.0.3) and R-studio (version 0.98.1091).

Differential expression of transcripts

Prior to computation of differentially expressed transcripts ('ANOVA testing for differences'), genes with less than five (non-normalized) read counts in all samples were excluded from analyses. Next, data normalization factors were calculated that are incorporated in further analyses by the weighted trimmed mean of M-values ("TMM") method (Robinson and Oshlack, 2010). After fitting of negative binominal models and both common, tagwise and trended dispersion estimates were obtained, differentially expressed transcripts were determined using a generalized linear model (GLM) likelihood ratio test (McCarthy et al., 2012). To minimize the effect of batch effect, resulting in possibly false positive results, 'sequencing batch' was included as a covariate in the GLM likelihood ratio test design matrix. All steps are implemented in the R-package edgeR (version 3.8.5) (Robinson et al., 2010). To determine differentially expressed transcripts, we only focused on transcripts with expression levels of logarithmic counts per million (LogCPM) > 3. Genes located on the Y-chromosome were omitted from all analyses, except from the gender-clustering

analysis, to exclude the effect of gender-specific mRNAs. Unsupervised hierarchical clustering was performed by Ward clustering and Pearson distances. Non-random partitioning, and corresponding p value, of unsupervised hierarchical clustering was determined using a Fisher's exact test.

Correlation matrices

The correlation of mRNA sequencing data from healthy donors and TEPs was determined using the mean counts per million (CPM)-normalized Log_2-transformed read counts per condition (healthy donors, TEPs). Pearson correlation coefficient was calculated by the 'cor'-function in R (package 'stats'). Red and blue dots, representing significantly increased and decreased transcripts, respectively, as determined by differential expression calculations, were superimposed on the graph. Pearson correlation coefficient of the sequenced platelet RNA samples with profiles of purified platelets, bone marrow megakaryocytes and circulating immune cells was determined using publicly available datasets. mRNA sequencing data of two, four, and four healthy donor platelet preparations, respectively, were obtained from Rowley et al. (Supplemental Table 6) (Rowley et al., 2011), Bray et al. (Supplemental Table S2A) (Bray et al., 2013), and Kissopoulou et al. (Supplemental Table 3) (Kissopoulou et al., 2013). The latter dataset was subdivided in a sample prepared using SMARTScribe Oligo-dT-primed cDNA synthesis and amplification (Sample S0), and ribosomal RNA depleted preparation (Sample S1-3) (Kissopoulou et al., 2013). Affymetrix microarray expression data (mean Log-intensities) of 154 healthy individuals was obtained from Simon et al. (Supplemental Table 2) (Simon et al., 2014). Four megakaryocyte mRNA sequencing profiles were obtained from Chen et al. (samples C006NSB1, C07002T4, C07015T4 and C12001RP2) (Chen et al., 2014)

and mRNA sequencing data from purified immune cells (i.e. granulocytes, NK-cells, B-cells, monocytes, memory, CD4, and CD8 T-cells) was obtained from Hrdlickova et al. (GSE62408) (Hrdlickova et al., 2014). Of note, expression data of four megakaryocyte datasets by Chen et al. and four healthy donors by Bray et al. were merged to a single expression value by computing the mean value per annotated gene. Of the individual datasets, the detected genes - and corresponding expression values - were converted to Ensembl gene annotations version 75, used for mapping of the platelet mRNA sequencing data. Remaining Ensembl annotations with no gene expression value were set at zero expression. All expression values were converted to Log_2-transformed RPKM values (except Simon et al. of which average Log_2 intensities was used, as provided by the authors). Following, all datasets were merged in a single datasheet, genes with zero expression in at least one dataset were excluded from analysis and sample gene expression correlations were computed by the 'cor'-function in R.

DAVID Gene Ontology (GO) analysis

DAVID GO was performed on the 23th of June 2015, using the online accessible DAVID database (http://david.abcc.ncifcrf.gov, version 6.7). Statistically significantly upregulated and down regulated ensemble gene IDs were assessed for enrichment in the following GO databases; GOTERM_BP_FAT, GOTERM_CC_FAT, GOTERM_MF_FAT, BBID, BIOCARTE, and KEGG_PATHWAY. GO terms with a FDR < 0.001 were reported.

Plasma DNA and platelet RNA amplicon sequencing (Amplicon-Seq)

Total RNA isolated from platelets was converted into cDNA by qScript (Quanta Biosciences). Amplicons covering the mutant hotspot regions were generated from platelet cDNA or plasma DNA using high-fidelity enzymes (Phusion HF; New England Biolabs). Each amplicon contains a 14 nt adapter sequence containing a nicking restriction enzyme site (Nb.BsrD1; New England Biolabs) enabling generation of unique 5'- and 3'- eight base pair sticky ends at each amplicon. After Nb.BsrD1 digestion, hairpin adaptor-barcode sequences with complementary ends were ligated to the purified amplicons by T4 DNA ligase (Invitrogen). The resulting circular DNA was subsequently amplified with primers to generate the *KRAS* (exon 2), or *EGFR* (exon 20 and 21) amplicons with adaptors and unique barcodes for the sequencing run. Quality control and quantitation of the amplicons was performed using the Bioanalyzer 2100 with DNA High Sensitivity chip or DNA 1000 chip (Agilent), after which up to 20 to 24 different amplicons were mixed in equimolar ratios to generate the amplicon library pool. The library pool was diluted to a molarity of 2 - 8 nM and again quality control was performed using a DNA High Sensitivity chip or DNA 1000 chip. After pooling of the library the standard Illumina protocol for the MiSeq sequencer was followed. The loading molarity was 6 pM or 9 pM, and the PhiX spike-in was 50% for every run. The runs were 2x150 cycles paired-end using the version 2 Reagent Kit system. Raw reads were trimmed and quality control was performed. Reads were mapped to the human reference genome (hg19) by Bowtie (v.1.1.2) while permitting three mismatches in the 70 nt seed region, unmapped reads were removed, and single nucleotide mutant variants were selected. Samples with less than 100,000 total read counts were excluded from analyses (i.e 4 out of 98 sequenced plasma samples and 11 out of 152 sequenced TEP samples). Detected

variants compared to the reference wild-type code included codon 12 (G12A, G12C, G12D, G12R, G12S and G12V) and codon 13 (G13D, G13C) of exon 2 of *KRAS* and exon 20 (T790M) and exon 21 (L858R and R861Q) of *EGFR*. Read counts were normalized for total number of reads per sample and corrected for intra-experimental variation. Per mutational variant, robust z-scores using the mean absolute deviation (MAD; robust Z = (x – m) / (1.4825 * MAD), in which *x* is the score of a sample and *m* is the median of the healthy donor cohort) were calculated and detected variants with a robust z-score of more than four standard deviations above the mean z-score of the healthy donor cohort were scored as positive. Furthermore, to take the error rate of the Phusion HF PCR enzymes into account, the ratio of specific mutant variant reads per wild-type reads had to exceed 1/5,000. Also, 'hypermutant' samples, defined as more than three mutant variants in KRAS, were excluded from analysis (i.e. 1 out of 235 analyzed samples). A cohort of 30 (KRAS) and 21 (EGFR) healthy donors plasma DNA and platelet RNA served as a control population and were included in the reported data as *KRAS* or *EGFR* wild-type individuals. Kappa statistics (Cohen's kappa coefficient for interrater agreement) and corresponding 95% confidence intervals were used to determine tissue-concordance.

SUPPLEMENTAL REFERENCES

Anders, S., Pyl, P.T., and Huber, W. (2014). HTSeq A Python framework to work with high-throughput sequencing data (Cold Spring Harbor Labs Journals).

Andrews, S., and others (2010). FastQC: A quality control tool for high throughput sequence data. Ref. Source.

Bolger, A.M., Lohse, M., and Usadel, B. (2014). Trimmomatic: a flexible trimmer for Illumina sequence data. Bioinformatics *30*, 2114–2120.

Dobin, A., Davis, C.A., Schlesinger, F., Drenkow, J., Zaleski, C., Jha, S., Batut, P., Chaisson, M., and Gingeras, T.R. (2013). STAR: ultrafast universal RNA-seq aligner. Bioinformatics *29*, 15–21.

McCarthy, D.J., Chen, Y., and Smyth, G.K. (2012). Differential expression analysis of multifactor RNA-Seq experiments with respect to biological variation. Nucleic Acids Res. *40*, 4288–4297.

Robinson, M.D., and Oshlack, A. (2010). A scaling normalization method for differential expression analysis of RNA-seq data. Genome Biol. *11*, R25.

Robinson, M.D., McCarthy, D.J., and Smyth, G.K. (2010). edgeR: a Bioconductor package for differential expression analysis of digital gene expression data. Bioinformatics *26*, 139–140.

Rolf, N. (2005). Optimized Procedure for Platelet RNA Profiling from Blood Samples with Limited Platelet Numbers. Clin. Chem. *51*, 1078–1080.

Wang, L., Wang, S., and Li, W. (2012). RSeQC: quality control of RNA-seq experiments. Bioinformatics *28*, 2184–2185.
